# Instruction in Tropical Medicine

**Published:** 1904-09-03

**Authors:** 


### Instruction in Tropical Medicine.

The historian of the future whose duty it shall be to record the position and achievements of medical science in the latter part of the nineteenth century, will certainly not omit to take note of the fact that then and there arose an active movement of scientific investigation into the causes of the diseases peculiar to tropical latitudes. And it will hardly be regarded as remarkable that in that movement Great Britain, with her vast foreign possessions, should have taken a leading part. It may certainly be taken for granted that whatever ultimate developments may wait on the study of tropical medicine the names of Patrick Manson and Ronald Ross will be recorded amongst those who provided the initial impetus and inspired the early direction and plan. As in all great movements there was for the pioneers a long period of obscure and unrecognised labour. But so thorough was this in its method, and so fruitful and far reaching in its results, that it may already claim a universal appreciation and recognition. One of the most practical testimonies in this direction is to be found in the generally admitted necessity for a special training, over and above the provisions of the ordinary medical curriculum, for those who propose to practise their profession in tropical countries. Not only is this demand an essential official condition of appointment to the Imperial Colonial Service but it is everywhere recognised as applicable to all who are called by duty or inclination to professional work in the warmer latitudes. The immediate claim upon these, as upon other medical practitioners, is the efficient care of the individual patients under their charge, and this, in view of recent developments, needs special knowledge and special training. But the preventive aspect of tropical medicine has now assumed very large proportions, and has both a humane and an imperial relationship; and yet again, it has become obvious that to the efficiently equipped student, and only to him, the diseases of warm climates offer opportunities for original research rich in the promise of great scientific and practical reward. It is these three aspects of the subject—cure, prevention, discovery—which must rule any efficient scheme of instruction in tropical medicine, and it may at once be said that there are in this country two institutions which fully recognise this need, namely the London and the Liverpool Schools of Tropical Medicine.

### The London School of Tropical Medicine.

The London School is situated near the Royal Victoria and Albert Docks, E., and is connected with the Branch Hospital of the Seamen's Hospital Society. The position of the school and hospital ensures a constant and abundant supply of clinical material in the shape of patients of various nationalities serving as seamen on ships entering the port. Hence acute cases of many diseases peculiar to the tropics can always be studied, and adequate provision is made for clinical instruction by the hospital staff. The laboratory course occupies necessarily a prominent part of the curriculum and includes daily practical teaching in the use of the microscope, the examination of the blood, and the recognition of the various parasites which are known to play so prominent a part in the production of tropical diseases. The full curriculum extends over three months and the inclusive fee is 16 guineas. In each year there are three sessions, beginning on October 1st, January 15th, and May 1st, respectively. Provision is made for the accommodation of a certain number of resident students for whom, also, board is provided. This is a great advantage, as the curriculum is a very full one, and those who wish to pursue it thoroughly will be well advised to go into residence. Lastly, a word may be said of the help afforded by daily tutorial teaching given by a capable and experienced medical tutor. Applications for admission to the school should be made as early as possible; it is not always possible to receive all who desire to attend.

### The Liverpool School of Tropical Medicine.

The Liverpool School conducts its laboratory work at University College and its courses of clinical instruction at the Royal Southern Hospital, where a special ward is allotted to the reception of cases of tropical disease. At so important a port there is, of course, always a large seafaring population, and thus clinical opportunities are well represented. The fee for a full course (lasting about two months) is

10 guineas. A hall of residence is attached to the school.

It is now competent, for those who desire it, to obtain a University Diploma in Tropical Medicine, and this may be taken either at Cambridge oı Liverpool. At Cambridge the fee is 6 guineas, and the examination is held in August. The Liverpool examinations are held three times a year, and the fee is 5 guineas. The University of Edinburgh also gives a special certificate in diseases of tropical climates. In all instances, both teaching and examinations are open to women as well as to men. At Liverpool there is a short course given to qualified nurses; the fee is 1 guinea.

